# Replica exchange molecular dynamics simulation of the coordination of Pt(ii)-Phenanthroline to amyloid-β†

**DOI:** 10.1039/c9ra04637b

**Published:** 2019-10-30

**Authors:** Matthew Turner, Shaun T. Mutter, Oliver D. Kennedy-Britten, James A. Platts

**Affiliations:** School of Chemistry, Cardiff University Park Place, Cardiff CF10 3AT UK platts@cardiff.ac.uk +44(0)-2920-874950

## Abstract

We report replica exchange molecular dynamics (REMD) simulations of the complex formed between amyloid-β peptides and platinum bound to a phenanthroline ligand, Pt(phen). After construction of an AMBER-style forcefield for the Pt complex, REMD simulation employing temperatures between 270 and 615 K was used to provide thorough sampling of the conformational freedom available to the peptide. We find that the full length peptide Aβ42, in particular, frequently adopts a compact conformation with a large proportion of α- and 3,10-helix content, with smaller amounts of β-strand in the C-terminal region of the peptide. Helical structures are more prevalent than in the metal-free peptide, while turn and strand conformations are markedly less common. Non-covalent interactions, including salt-bridges, hydrogen bonds, and π-stacking between aromatic residues and the phenanthroline ligand, are common, and markedly different from those seen in the amyloid-β peptides alone.

## Abstract 2

Replica exchange molecular dynamics are used to explore the conformational freedom of amyloid-βbound to Pt(phenanthroline), highlighting important differences in secondary and tertiary structure from the metal-free peptide.

## Graphical abstract

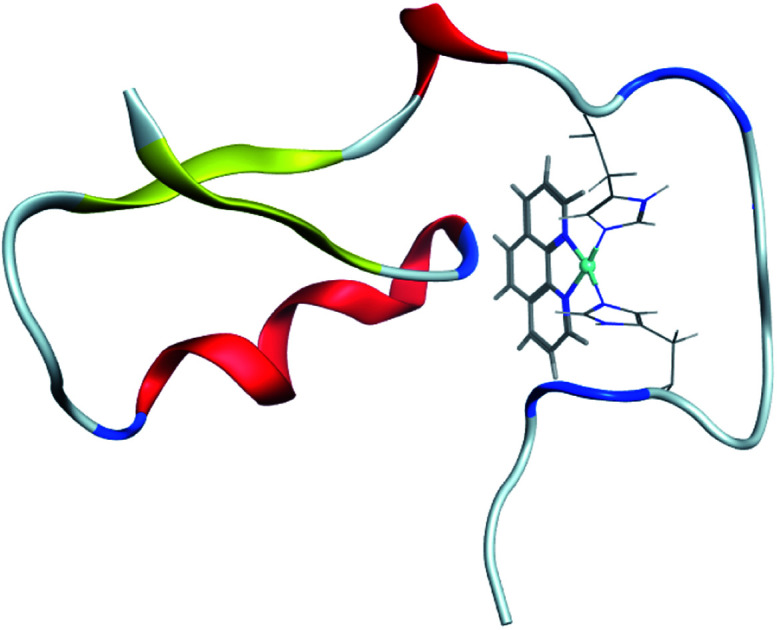

## Introduction

Alzheimer's disease (AD) is a devastating neurodegenerative condition that poses major healthcare challenges. Significant hallmarks of AD include the death of neurons and their connections in addition to the presence of insoluble plaques and neurofibrillary tangles.^1^ The amyloid cascade hypothesis^2–7^ suggests that aggregation of the amyloid-β (Aβ) peptide into soluble oligomers, fibrils and plaques is the main driver of AD, whereas the metal ion hypothesis^8–22^ suggests that disruption of metal ion homeostasis promotes Aβ aggregation and onset of AD.

Barnham *et al.*^23–26^ demonstrated that Pt(phenanthroline) and related complexes are able to bind Aβ and inhibit fibril formation and Aβ toxicity. They also noted that cisplatin has no effect on this process, indicating that the planar aromatic ligands confer some specificity for Aβ to the platinum complexes. In addition, the aromatic ligands form stabilising noncovalent interactions through π–π stacking with aromatic residues such as Phe4, Tyr10, and/or His6, 13 or 14. Later work by Ma *et al.*^27,28^ determined that the Pt(phen) complex preferentially binds to His6 and His14 in Aβ16. EPR data indicated that Cu^2+^/Zn^2+^ remain bound to Aβ in the presence of Pt(phen), but that the copper/zinc binding sites are altered.

Despite the promise of Pt complexes as potential treatments for AD, detailed structural data on their effect on Aβ is relatively scarce. Streltsov *et al.*^29^ used a combination of EXAFS and density functional theory (DFT) to confirm binding through His residues, while our group has used DFT, semi-empirical and molecular mechanics methods to examine Pt binding to model peptides.^30–32^ The intrinsically disordered nature of Aβ means that such studies struggle to sample the full conformational flexibility of the peptide. To address this possible shortcoming, and to provide further details of possible Pt(phen)–peptide interactions and the effect of metal coordination on peptide structure at a molecular level, we performed replica-exchange molecular dynamics (REMD) simulations of the platinum complex interacting with both the N-terminal Aβ16 fragment and complete Aβ42 peptides. REMD is a popular means of improving the reliability and scope of MD simulations, and has been used previously to enhance sampling of possible conformations of the intrinsically disordered Aβ peptide.^33–38^ Application to metal binding to Aβ are rare, but one recent study showed how Hamiltonian replica exchange molecular dynamics (H-REMD) enhances sampling of copper–Aβ complexes.^39,40^ Gaining understanding of how these complexes interact with the Aβ peptide may help explain the effect of Pt-coordination on conformational flexibility of Aβ, and hence provide insight on their anti-AD activity.

## Computational methods

**Scheme 1:**
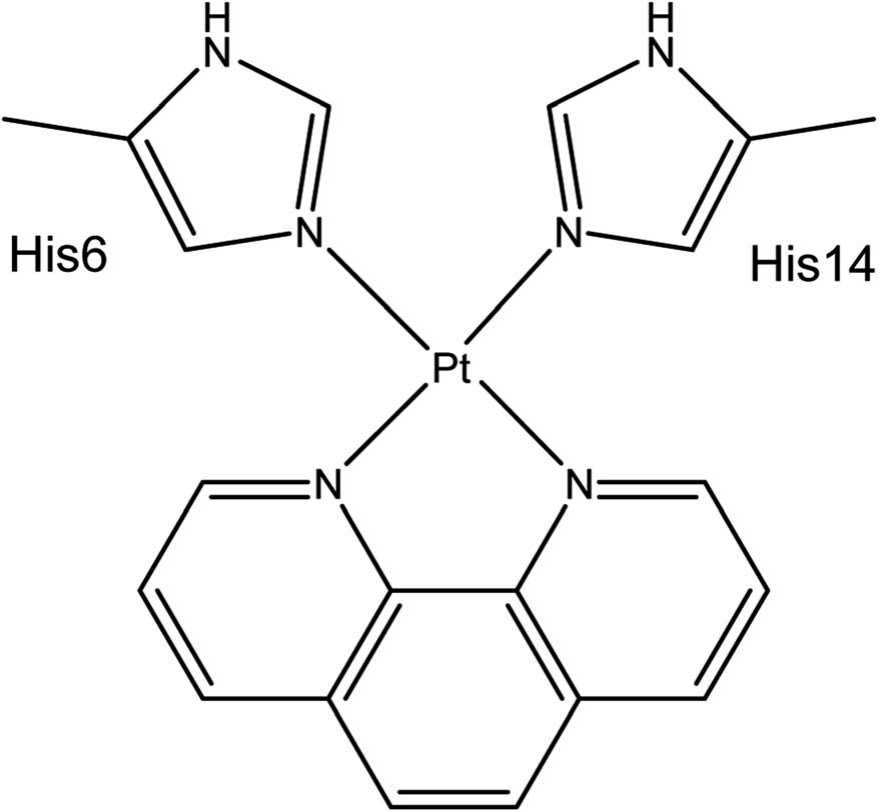
Coordination mode of Pt(phen)-Aβ16.

Aβ peptides were constructed in extended conformations^41^ with appropriate residue protonation states for physiological pH. Pt(ii) was coordinated *via* His6 Nε and His14 Nε, as identified by experimental and computational work and shown in Scheme 1. Structures were subjected to short LowMode^42^ conformational searches to obtain reasonable starting structures.

REMD simulations were performed using the AMBER16 package.^43^ The AMBER ff14SB forcefield parameter set^44^ was used to model all standard amino acid residues; organic ligands were modelled using GAFF.^45^ The bonded MCPB.py^46^ approach was used for Pt(ii). Here metal bonding parameters are derived from B3LYP/6-31G(d) frequency calculations and RESP charges for the metal-coordinating regions were obtained from the same level of theory using Gaussian09.^47^ Both simulations exponentially spanned the temperature range between 270 and 615 K: for Aβ16, 10 replicas were used, while for the larger Aβ42 16 replicas were used. REMD simulation parameters were obtained using the online REMD temperature generator.^48^ For each replica, 110 ns of REMD simulation was performed and 110 000 conformations collected. The first 10 ns was treated as equilibration, and the last 100 ns was used for data analysis, following the procedure used by Yang & Teplow.^33^

The Generalised Born solvation model^49–51^ was used to solvate all Pt(ii)-Aβ systems, since the use of implicit solvent model enhances conformational sampling.^52^ REMD simulations were carried out in the NVT ensemble, using a Langevin thermostat to control temperature. Exchange between replicas was attempted every 2 ps. SHAKE was used to constrain bonds to hydrogen. Simulations were performed using a 2 fs integration time step. Analysis of the trajectories was performed using CPPTRAJ v16.16 (ref. 53) and VMD 1.9.3.^54^ Secondary structure assignment was made using the DSSP algorithm^55^*via* CPPTRAJ. Salt bridges were defined as any contact distance of less than 3.2 Å between oxygen and nitrogen atoms in charged residues. Ramachandran maps were made using MDplot.^56^ Clustering was performed using the DBSCAN algorithm Ester *et al.*,^57^ using backbone RMSD as the metric, with MinPoints = 5 and *ε* = 0.8.

## Results and discussion

An AMBER style forcefield for Pt(phenanthroline) bound to Aβ through histidines was constructed using MCPB.py from results of B3LYP/6-31G(d) optimisation, frequency and electrostatic potential. This resulted in bonding parameters (reported as [*r*_0_, *k*] in units of Å and kcal mol^−1^ Å^−2^) of [2.06, 107] for Pt–N_phen_ and [2.05, 127] for Pt–N_His_, indicating slightly stronger bonds to His than to phen. Angle parameters ([*θ*_0_, *k*] in ° and kcal mol^−1^ deg^−2^) were [81, 169] for N_phen_–Pt–N_phen_, [89, 150] for N_His_–Pt–N_His_, [95, 158] for N_phen_–Pt–N_His_ in *cis*-arrangement, and [176, 167] for *trans*-N_phen_–Pt–N_His_, reflecting the distortions from purely square planar values required by the bidentate phenanthroline ligand. Non-bonded parameters for Pt were charge = +0.027 *e*, *ε* = 0.0031 kcal mol^−1^ and *σ* = 1.266 Å. Full details of all forcefield parameters and the resulting coordination geometry around Pt are available as ESI (Tables S1 and S2).†

Using this forcefield, REMD with 10 and 16 replicas for Pt-Aβ16 and Pt-Aβ42, respectively, covering temperatures ranging from 270 to 625 K ran successfully, with exchange frequencies averaging 0.146 and 0.196 across all frames. Root mean square deviation (RMSD) from the energy minimised starting point reached maximal values of 9.5 and 28.9 Å for Pt-Aβ16 and Pt-Aβ42, respectively. Similarly, the radius of gyration (*R*_g_) reached maximal values of 11.9 and 32.3 Å. Averaged over the final 100 ns for all replicas at all temperatures, mean RMSD was 5.0 and 13.6 Å, while mean *R*_g_ was 9.3 and 17.2 Å. For both peptides, mean and maximum RMSD and *R*_g_ are significantly larger for the entire REMD ensemble than for the trajectory closest to 300 K (*vide infra*), indicating that the use of elevated temperatures in the REMD ensemble ensured increased conformational sampling over MD at a single temperature.

Table 1 reports mean and standard deviation of RMSD values for trajectories closest to 300 K for both Pt-Aβ16 (299.3 K) and Pt-Aβ42 (304.4 K). In both cases, the standard deviation of around 1.5 Å indicates simulations reach a stable set of configurations, and the maximum value reached is considerably less than for the entire REMD ensemble, particularly in the longer peptide where the maximum value in the 300 K trajectory is around half the maximum total value. The relatively large force constants for Pt–N bond stretching leads to stable coordination of Pt(phen) to Aβ (Table S1†), with Pt–N_His_ distances both averaging 2.06 ± 0.06 Å (ESI Table S2†).

**Table 1:** Pt-Aβ16 and Pt-Aβ42 RMSD statistics from room temperature REMD trajectory (Å)

	RMSD		*R*_g_	
	Pt-Aβ16	Pt-Aβ42	Pt-Aβ16	Pt-Aβ42
Ave	3.63	10.55	9.17	11.67
SD	1.31	1.65	0.68	1.57
Max	7.63	15.51	11.14	26.31

Details of *R*_g_ for 300 K simulations of Aβ16 and Aβ42 are also shown in Table 1. On average, Pt-Aβ16 adopts a relatively compact conformation at room temperature, with average *R*_g_ of 9.2 Å. For comparison, Aβ16 in an extended conformation has *R*_g_ = 17 Å, and as α-helix *R*_g_ = 9.2 Å, while complexes with Cu, Fe and Zn have average *R*_g_ between 7.2 and 7.6 Å.^58^ The larger *R*_g_ for these complexes reflects the size of the phenanthroline ligand (*R*_g_ = 2.3 Å): subtracting this from the overall *R*_g_ indicates Pt induces conformations of Aβ16 that are similar in compactness to other transition metals, despite coordinating to only two residues rather than four or five. Aβ42 also adopts a compact conformation, its overall average *R*_g_ of 11.6 Å only slightly larger than that for the free peptide (9.6 Å^59^), when the size of the Pt(phen) is taken into account. The possible role of non-covalent interaction in maintaining such compact conformations is explored below.

The principal moments of the *R*_g_ tensor provide information on the shape adopted throughout the trajectory. Full data are provided in ESI (Table S3),† which show that on average both peptides adopt prolate spheroidal geometry, with two small and one large eigenvalues. These can be used to construct shape descriptors,^60^ as has been done recently for Aβ oligomerisation pathways,^61^ in particular anisotropy that varies from 0 (ideal linear chain) to 1 (fully symmetric). These values are on average 0.252 for Pt-Aβ16 and 0.245 for Pt-Aβ42, showing that both peptides are highly anisotropic. The *R*_g_ tensors also highlight the sampling of conformations that results from use of REMD, for Pt-Aβ42 in particular, where the largest value of the largest eigenvalue reaches 650 Å^2^, a value that corresponds to an almost fully extended conformation (Fig. S2†).

**Fig. 1:**
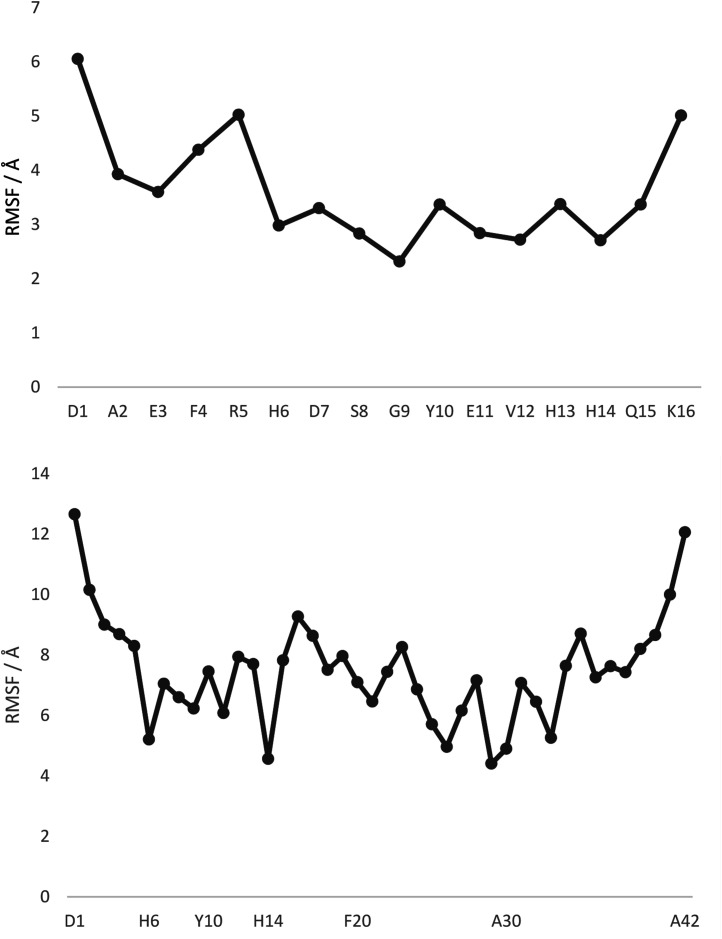
RMSF values, by residue, from 300 K REMD trajectories. Pt is bound to H6 and H14 in both cases, with selected other residues shown for clarity.

Fig. 1 displays root mean square fluctuation (RMSF) data by residue for both peptides at room temperature (numerical data is available in Table S4†). It is apparent that both N- and C-termini are highly flexible, while residues bound to Pt (His6 and His14) are relatively immobile (RMSF 3.0/2.7 Å in Pt-Aβ16, 5.2/4.6 Å in Pt-Aβ42). This is not limited to the metal-coordinating residues: residues from Asp7 to His13 also exhibit relatively low RMSF. Flexibility is high in the central hydrophobic region (CHC), *i.e.* Leu17 to Ala21, falls between Gly25 and Ile32 with particularly low value for Gly29, then rises again toward the C-terminus. Overall statistics for RMSF by amino acid are reported in Table 2, showing that the longer peptide is markedly more mobile. Table S4† also reports RMSF values for the Pt atom and the phen ligand, which are again notably larger for Pt-Aβ42. The origin of this behaviour, and its consequences for the conformations adopted by the full peptide, are explored in more detail below.

**Table 2:** RMSF statistics for 300 K trajectories (Å)^a^

	Pt-Aβ16	Pt-Aβ42
Mean	3.61	7.49
SD	1.03	1.77
Min	2.32	4.41
Max	6.06	12.66

a C_α_–C_α_ distances in Pt-Aβ16 and Pt-Aβ42 are used to interrogate the tertiary structure of the peptides, as represented in the contact maps in Fig. 2. In the shorter peptide, the longest inter-residue distances occur for Asp1, with values in excess of 20 Å for residues Val12 through Lys16. Indeed there is little structure in the contact map: close contact between Pt-binding residues 6 and 14 is apparent, while the sequence between these is also restricted to relatively short distances. The longer peptide exhibits more structure: Asp1 again has the longest distances, most notably to amino acids in the CHC and the extreme C-terminus. Longer distances are also found for the sequences Ser8-Tyr10 with Asp23-Lys28. Short contact is found for His6 with His14 and the residues between them, and also between Phe19-Ala21 with Gly29-Gly33.

**Fig. 2:**
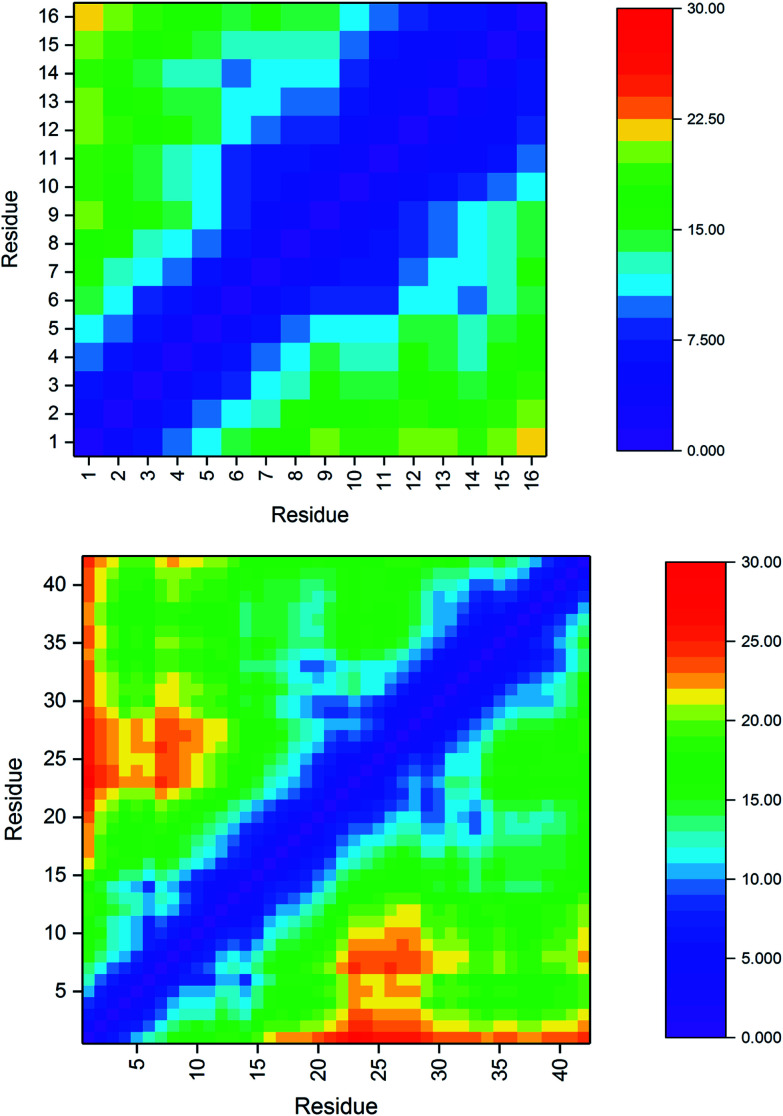
Pt-Aβ16 and Pt-Aβ42 *C*_α_ distance matrices (Å), demonstrating close contacts (blue) between His6 and His14 due to Pt coordination, as well as ween Phe19-Ala21 with Gly29-Gly33 in the larger peptide.

**Fig. 3:**
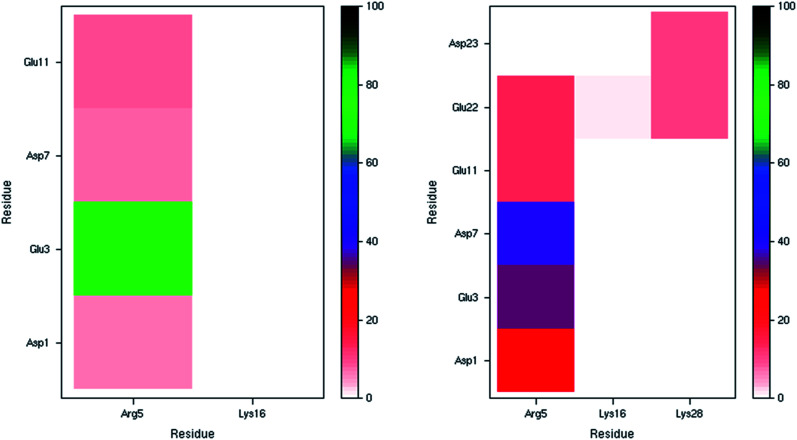
Salt bridge occurrence (%) from 300 K simulations indicating just one prevalent interaction in the smaller peptide (left), and two in the larger one (right).

The incidence of salt bridge interactions in Pt-Aβ16 and Pt-Aβ42 are summarised in Fig. 3 (numerical data in Table S5†). In the shorter peptide, C-terminal Lys16 forms almost no salt bridges (occupancy < 0.5% of frames), whereas Arg5 forms these stabilising interactions much more frequently. Glu3-Arg5 is especially prevalent, occurring in 76% of frames, while interactions with Asp1, Asp7 and Glu11 are present but rarer (between 5 and 10% of simulation time). The low occupancy of Asp1 salt bridges is in agreement with the large RMSF and C_α_ contact distances noted above. Furthermore, the low incidence of both the Asp7-Arg5 and Glu11-Arg5 interactions may be as a result of the large Pt(phen) system binding to His6 and His14 that flank these charged sites. In Pt-Aβ42, Arg5 again engages in most salt bridges, most notably with Glu3 and Asp7, but also Asp1, Glu11 and Glu22. Lys16 again engages in hardly any interactions, while Lys28 interacts only with Glu22 and Asp23, but only for around 10% of frames. The latter interaction is believed to play a role in the conformational changes that accompany aggregation of Aβ42 into fibrils;^62^ the ability of Pt(phen) to disrupt this may be an important aspect of its effect on peptide aggregation.

**Fig. 4:**
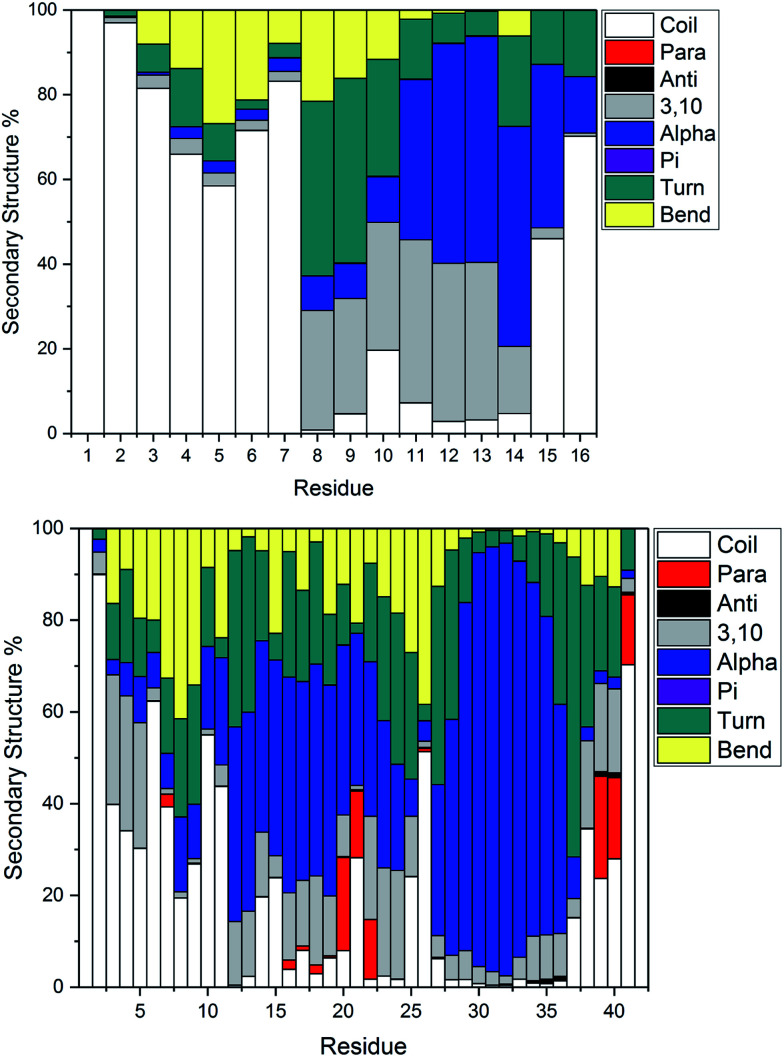
Average secondary structure percentage, indicating significant bend, turn and some helical content in the smaller peptide (top), with more helical and some β-strand content in the larger peptide (bottom).

Fig. 4 illustrates the per-residue peptide secondary structure of Pt-Aβ16 and Pt-Aβ42. N-terminal residues in Aβ16 adopt coil conformations most frequently, with small amounts of turn and bend, while the region between the metal-binding sites (His6-His14) adopts helical structure more frequently. In particular, α-helical content is especially common from Glu11-Gln15 (up to 53% of simulation), while 3,10 helices are prevalent between Ser8-His14 (up to 37%). This region also contains significant amounts of turn and bend (*ca.* 60%) structures centred on Ser8-Gly9. C-terminal Gln15 and especially Lys16 are less structured, although some α-helix structure is retained here.

Pt-Aβ42 exhibits more varied secondary structure: only the very terminal residues Asp1, Ala2 and Ala42 are predominantly coil in form, whereas helical structures are apparent throughout the remainder of the sequence. This is mostly 3,10 in character for Glu3-Arg5, but α-helix accounts for over 50% of conformations in Val12-Val24, and over 80% of Lys28-Val36. Approximately 20% of conformations of Phe20-Glu22 and Val39-Ile41 adopt parallel β-sheet geometry, indicating that these residues form through-space contacts for a significant portion of the trajectory. Turn/bend forms are also present, notably for Ser8-Gly9 (62%), Val24-Gly25 (53%) and Gly37-Gly38 (57%). It is clear from this that Pt-coordinated Aβ forms numerous and stable helical structures, rather more than is found for other metals.^59^ It may be that this preference for helical structure is related to the known inhibition of fibril formation induced by Pt(phen), for example by reducing the propensity for this over formation of the β-strands that are thought to act as the seeds for aggregation.^63^

Secondary structure percentages for the platinum-coordinated peptides adopted at 300 K are shown in Table 3, combining α/π/3,10 helices, parallel/antiparallel sheets, and turn/bend/coil into single measures. As expected for an intrinsically disordered peptide, including sampling from high temperatures in REMD, random coil/turn/bend conformations form the largest category. These data show that Aβ16 has almost one-third of residues in helical orientation, but almost no β-sheet, while Aβ42 has significantly more helical content as well as a small amount of β-sheet.

**Table 3:** Pt-Aβ16 secondary structure percentages

	Helix	Sheet	Other
Pt-Aβ16	32.5	0.01	67.4
Pt-Aβ42	42.8	2.8	54.4

**Fig. 5:**
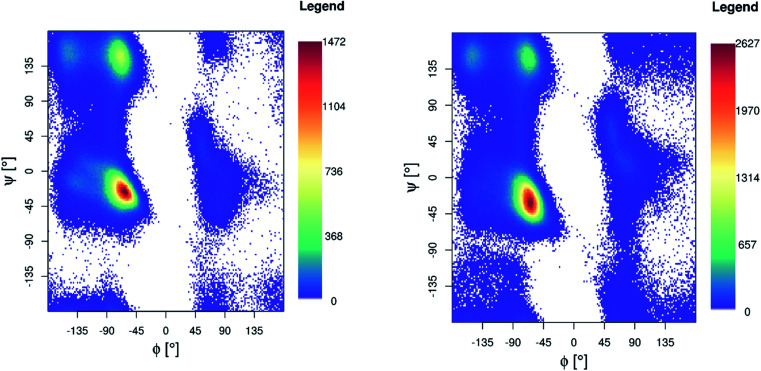
Ramachandran plot for Pt-Aβ16 (left) and Pt-Aβ42 (right) 300 K REMD simulation.

Ramachandran maps for both Pt-Aβ16 and Pt-Aβ42 (Fig. 5) are dominated by right-handed α-helical conformations, centred on (−60°, −40°), with smaller but noticeable populations of poly-proline II (−75°, 150°) and β-sheet (−150°, 160°) structure, as well as small amounts of left-handed helical structures. Following the nomenclature of Hollingsworth and Karplus,^63^ we see little evidence for γ structures, but γ′ at (−80°, +80°) is populated as part of the “bridge” region.

Table 4 reports statistics relating to the number of observed hydrogen bonds: Pt-Aβ16 has, on average, slightly less than five hydrogen bonds per simulation frame, the large standard deviation indicating that these interactions are fluxional, as shown by configurations with no hydrogen bonds as well as others with as many as fourteen. Only 6 hydrogen bonds persist for more than 10% of simulation time, the most prevalent of these, present for 34% of the trajectory, links His14 Nδ–H with backbone O of Glu11, which also H-bonds to backbone of Gln15 (18% of frames). Oε of Glu3 interacts with backbone N–H of Phe4 for 33% of simulation. For Pt-Aβ42 the values are larger but the pattern is similar: the average of 11.7 ± 3.0 incorporates a range of between 25 and 0 hydrogen bonds. 26 interactions are present for at least 10% of the simulation, including Glu11 Oε to His14 Nδ–H (48%), Gly29 backbone O to Gly33 N–H (37%), and Glu11 backbone O to Gln15 backbone N–H (23%). The latter are among numerous *i*+4 → *i* backbone–backbone interactions associated with α-helix structure are observed (23 with at least 2% occupancy, Table S7†), mainly in the CHC and C-terminus. 20 *i*+3 → *i* hydrogen bonds, characteristic of 3,10-helices, are also observed along with 3 *i*+5 → *i* interactions that are characteristic of π-helical structures, mainly in the C-terminus (see Table S7† for full details).

**Table 4:** Statistics of hydrogen bond occupation

	Pt-Aβ16	Pt-Aβ42
Ave	4.7	11.7
SD	1.8	3.0
Min	14	25
Max	0	0

As well as hydrogen bonds, stacking interactions between peptide and phenanthroline ligand have been suggested to play a role in specific binding and disruption of the structure and aggregation of Aβ. Potential interactions were sought by monitoring the shortest distance between Cα/Cβ/Cγ of each amino acid and any carbon atom of the ligand. Variance within these distances is large, but for Pt-Aβ16 Tyr10 and His13 stand out as being commonly found in proximity to the ligand, with average distances of 6.86 ± 3.43 Å and 7.16 ± 2.45 Å. 35% of frames contain at least one atom–atom distance of less than 5 Å between Tyr10 and phen, and 9% have such contacts with His13. The closest contact with Tyr10 consists of face-to-face π-stacking with phenanthroline, with distance between ring centroids = 3.55 Å and angle between planes of 0.18°. The closest contact with His13 also exhibits stacking with the ligand, albeit with larger separation and reduced co-planarity (distance = 3.81 Å, angle = 12.7°). In contrast, Phe4 only sporadically makes contact with the ligand, with less than 2% of frames within 5 Å, and average separation 14.91 Å.

In contrast, in Pt-Aβ42 only Phe4 forms persistent non-covalent interactions with phenanthroline within the room temperature REMD trajectory, with an average distance of 9.98 ± 4.61 Å, and 15% of frames within 5 Å. Tyr10 and His13 exhibit longer averages (14.5 and 11.0 Å) and fewer frames with close contact (<1%). The closest contact of Phe4 is also parallel π-stacking, with distance between ring centroids = 4.02 Å and angle between planes of 2.75°. It seems, therefore, that the more flexible nature of the full-length peptide leads to more transient stacking interactions than are observed in the truncated one alone. Such interactions are nevertheless present with all aromatic side chains, and may play a role in binding of Pt(phen) to the N-terminus as well as the conformations then adopted.

**Fig. 6:**
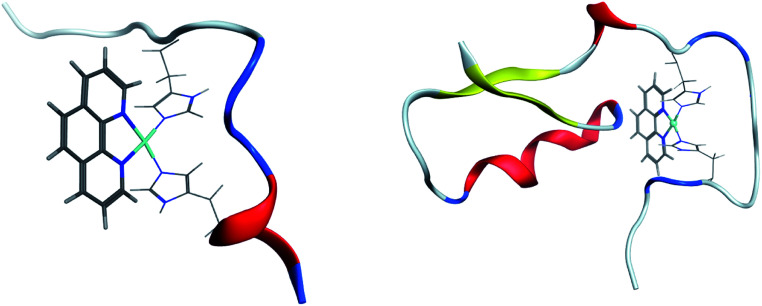
Representation of most populated clusters of Pt-Aβ16 and Pt-Aβ42, with Pt(phen) shown as solid lines, coordinated His as wireframe, and peptide backbone as ribbon (red = helix, yellow = sheet, blue = turn, white = coil).

Clustering of room temperature trajectories gave rise to sparsely populated clusters, with maximum population of 2.5% and 1% for Pt-Aβ16 and Pt-Aβ42, respectively. Representative structures of the most populated cluster for each are shown in Fig. 6. Firstly, this demonstrates the stability of the Pt(phen)(His)_2_ coordination mode, with imidazoles of His6 and His14 approximately perpendicular to the plane of Pt(phen). In common with the patterns noted above, Pt-Aβ16 is mainly coil in the N-terminus, with turn and a small helical element toward the C-terminus. This structure also shows evidence of the non-covalent interactions noted above, with both Tyr10 and His13 approximately 4.5 Å from phenanthroline in a slipped-parallel stacked arrangement. More secondary structural elements are apparent in Pt-Aβ42, although here again the N-terminus is relatively unstructured. Helical structure is observed for the sequence Lys28-Val36, along with an antiparallel β-sheet connecting Phe20-Asp23 with Val39-Ala42, separated by turns centred on Val12 and Asn27. The phenanthroline is located in a hydrophobic environment, with Leu34, Met35 and Val36 in particular located close to the π-system, but in this particular structure none of the aromatic residues discussed above are found within 5 Å of the ligand.

## Conclusions

Replica exchange molecular dynamics has been used to provide enhanced sampling of the conformational freedom available to the adduct formed between amyloid-β peptides and Pt(ii)phenanthroline. We report data both for the metal-binding N-terminal sequence (Pt-Aβ16) and for the complete peptide (Pt-Aβ42), each bound to Pt through His6 and His13. AMBER-style forcefields for these systems were developed using the MCPB approach for Pt, along with GAFF for the phenanthroline ligand. The success of REMD in enhancing sampling is demonstrated by exchange frequencies between simulations at different temperatures, ranging from 270 to 615 K, of between 0.15 and 0.2, and also by the fact that the entire ensemble includes structures that are much more extended than those visited by simulations at room/body temperature alone.

A key aspect of any such study is the suitability of the methods used, which in this case means the forcefield and solvent model. Parameters from Li and Merz's MCPB.py method for Pt and coordinated atoms, along with GAFF for phenanthroline ligand, reproduce known coordination geometry of Pt(ii) bound to two His residues. The ff14SB forcefield used for peptide here is the primary model in the AMBER suite, reported to yield improved secondary structure content in small peptides and NMR data for proteins in solution compared to previous versions. However, a recent report^64^ suggests it may be imbalanced when comparing folded and disordered proteins: we hope to explore the effect of different forcefields in future studies. Another issue may be the use of an implicit solvent model, chosen here for the enhanced sampling that it enables, but which might also lead to imbalance in intramolecular over intermolecular interactions. This approach is widely used in study of intrinsically disordered proteins (see for instance^65,66^ for recent examples). Moreover, the radius of gyration we report is in the range of values reported in literature, namely 9 to 13 Å with a mean of 11.4 Å,^67^ which lends confidence to the suitability of the approach we have taken.

Extracting trajectories only for the temperatures closest to 300 K (299 and 304 K for Pt-Aβ16 and Pt-Aβ42, respectively) allows us to assess the range of structures accessible to these systems at physiologically relevant temperatures. Both systems are found to be relatively compact: once the size of Pt(phen) is accounted for, radius of gyration is similar to Cu, Zn and Fe complexes. Secondary structure, particularly helical, is present for both peptides: the full-length Aβ42 especially has extensive α- and 3,10-helices, most notably in the metal-binding region and in the central hydrophobic core. A smaller but significant proportion of frames are found to be in β-strand form in residues toward the C-terminus. Of the possible salt bridges that may form within Aβ, relatively few are present for substantial portions of trajectories: Glu3-Arg5 is the most common in both systems. Asp23-Lys28, which has been implicated in fibril formation, is only populated for 10% of recorded frames.

Non-covalent interactions also play a role: hydrogen bonds, especially those associated with helical structures, are very common but also highly fluxional: in both cases, frames with no H-bonds at all are observed. Stacking interactions between aromatic residues and phenanthroline are also observed, especially for the shorter Pt-Aβ16, where His6 and Tyr10 make frequent contact with the ligand. In the larger peptide these residues are less commonly found in proximity with the ligand, whereas Phe4 is more commonly disposed in this way. Overall, direct contact between aromatic amino acids and the phenanthroline ligand is present in all simulations, and apparently plays a significant role in the ensemble of conformations adopted by the adducts.

It is instructive to compare the results obtained here with analogous data obtained for the free Aβ42 peptide by Yang and Teplow.^33^ We note that this used a different forcefield (parm99SB), so any comparisons can only be qualitative. Compared to that work, we find much more helical but notably less turn and β-strand content than in the free peptide. This is particularly evident around residues 10 to 20, for which the free peptide's α-helical content is less than half of that found here, while turn percentages for residues 23–26 reported to be as high as 70% in the free peptide are reduced to half or less of those values once Pt(phen) is bound. Tertiary structure is also affected: turn structures highlighted for the N-terminus of the free peptide are lost after Pt binding, as are weaker contacts between residues 6–8 with 23–28, although other contact between the CHC and residues 30–36 are common between both simulations. Although this is far from conclusive, the global effect on peptide conformation of Pt(phen) binding through just two N-terminal residues is striking, and may shed new light on the origin of its observed anti-aggregation effects.

## Data statement

Trajectories for room temperature simulations and coordinates of most populated clusters, in PDB format, have been deposited on Zenodo with DOI: 10.5281/zenodo.3250227.

## Author contributions

MT and JAP designed experiments; STM and MT carried out simulations; STM, MT, OKB and JAP analysed data; JAP and MT wrote the manuscript.

## Conflicts of interest

There are no conflicts to declare.

## Supplementary Material

RA-009-C9RA04637B-s001
