# Supplementary material for: Replica exchange molecular dynamics simulation of the coordination of Pt(ii)-Phenanthroline to amyloid-β
Source: RSC Adv. 2019 Oct 30;9(60):35089–97. doi: 10.1039/c9ra04637b (PMC9074135; doi:10.1039/c9ra04637b)
Supplement: RA-009-C9RA04637B-s001 [file RA-009-C9RA04637B-s001.pdf]

Electronic Supplementary Material (ESI) for RSC Advances.
This journal is © The Royal Society of Chemistry 2019

Pt REMD Supplementary data

Table S1: MCPB.py forcefield data

```
MASS
M1 195.08                       Pt ion
Y1 14.01         0.530          sp2 N in 5 memb.ring w/LP (HIS,ADE,GUA)
Y2 14.01         0.530          sp2 N in 5 memb.ring w/LP (HIS,ADE,GUA)
Y3 14.01         0.530          Sp2 N in pure aromatic systems
Y4 14.01         0.530          Sp2 N in pure aromatic systems

BOND
M1-Y3  107.2    2.0597      Created by Seminario method using MCPB.py
M1-Y4  107.3    2.0594      Created by Seminario method using MCPB.py
Y1-M1  127.3    2.0562      Created by Seminario method using MCPB.py
Y2-M1  127.3    2.0562      Created by Seminario method using MCPB.py

ANGL
CR-Y1-M1   138.28     126.69    Created by Seminario method using MCPB.py
CR-Y2-M1   136.04     126.68    Created by Seminario method using MCPB.py
CV-Y1-M1   141.21     126.20    Created by Seminario method using MCPB.py
CV-Y2-M1   139.67     126.19    Created by Seminario method using MCPB.py
M1-Y3-ca   169.55     120.57    Created by Seminario method using MCPB.py
M1-Y4-ca   169.59     120.57    Created by Seminario method using MCPB.py
Y1-M1-Y2   149.86      88.55    Created by Seminario method using MCPB.py
Y1-M1-Y3   159.53      95.41    Created by Seminario method using MCPB.py
Y1-M1-Y4   168.05     175.94    Created by Seminario method using MCPB.py
Y2-M1-Y3   166.27     176.00    Created by Seminario method using MCPB.py
Y2-M1-Y4   156.32      95.47    Created by Seminario method using MCPB.py
Y4-M1-Y3   168.87      80.58    Created by Seminario method using MCPB.py
```

Table S2: Pt-N distance statistics for Pt-Aβ16 (Å)

	Ave	SD	Min	Max
Pt-His6 N	2.062	0.058	1.771	2.352
Pt-His14 N	2.065	0.058	1.763	2.377

Table S3: Eigenvalues of the Rg-tensor for 300 K simulations (Å^2)

	Pt-Aβ16			Pt-Aβ42		
	Eig1	Eig2	Eig3	Eig1	Eig2	Eig3
Ave	9.50	19.14	55.90	18.24	32.49	97.99
SD	1.58	4.53	15.22	3.73	6.61	45.23
Max	18.96	40.58	101.56	34.77	86.65	649.76

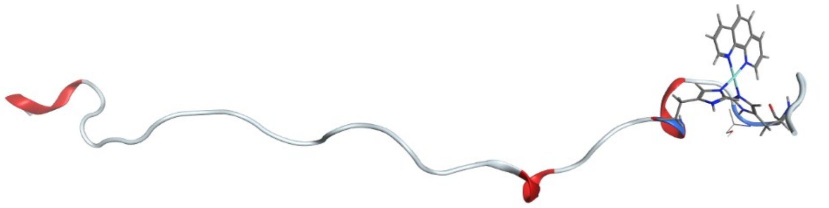

Figure S1: Structure of complex with Eig3 = 650 Å^2

Table S4: RMSF by residue

**1-16**			**1-42**				
**1**	6.0558		**1**	12.6565		**23**	8.2632
**2**	3.9262		**2**	10.1557		**24**	6.8617
**3**	3.5999		**3**	9.0047		**25**	5.7062
**4**	4.3778		**4**	8.6911		**26**	4.9609
**5**	5.0238		**5**	8.2984		**27**	6.1602
**6**	2.9798		**6**	5.2059		**28**	7.1622
**7**	3.3004		**7**	7.0491		**29**	4.4072
**8**	2.8331		**8**	6.6011		**30**	4.9004
**9**	2.3164		**9**	6.23		**31**	7.075
**10**	3.3683		**10**	7.4542		**32**	6.4524
**11**	2.8378		**11**	6.0822		**33**	5.2601
**12**	2.7178		**12**	7.9379		**34**	7.6428
**13**	3.3749		**13**	7.6998		**35**	8.708
**14**	2.7071		**14**	4.5638		**36**	7.2621
**15**	3.3672		**15**	7.8232		**37**	7.6283
**16**	5.01		**16**	9.2735		**38**	7.4299
**Pt**	3.8089		**17**	8.6368		**39**	8.2022
**phen**	5.5910		**18**	7.5109		**40**	8.6588
			**19**	7.9634		**41**	9.9994
			**20**	7.0989		**42**	12.0622
			**21**	6.4629		**Pt**	5.4906
			**22**	7.4429		**phen**	8.2088

Table S5: salt bridge occurrence (%)

Pt-Aβ16	Asp1	Glu3	Asp7	Glu11
Arg5	6.2	76.3	7.1	8.3
Lys16	0.1	0.4	0.1	0.3

Pt-Aβ42	Asp1	Glu3	Asp7	Glu11	Glu22	Asp23
Arg5	25.0	34.4	33.4	13.8	13.2	0.1
Lys16	0.0	0.0	0.0	0.1	1.3	0.5
Lys28	0.0	0.0	0.0	0.0	11.0	10.3

Table S6: Percentage secondary structure

Pt-Aβ16	Coil	Para	Anti	3,10	Alpha	Pi	Turn	Bend
1	100	0	0	0	0	0	0	0
2	97	0	0	1	0	0	1	0
3	81	0	0	3	1	0	7	8
4	66	0	0	4	3	0	14	14
5	58	0	0	3	3	0	9	27
6	72	0	0	2	3	0	2	21
7	83	0	0	2	3	0	3	8
8	1	0	0	28	8	0	41	22
9	5	0	0	27	8	0	44	16
10	20	0	0	30	11	0	28	12
11	7	0	0	38	38	0	14	2
12	3	0	0	37	52	0	7	1
13	3	0	0	37	53	0	6	0
14	5	0	0	16	52	0	21	6
15	46	0	0	3	39	0	13	0
16	70	0	0	1	13	0	16	0

Pt-Aβ42	Coil	Para	Anti	3,10	Alpha	Pi	Turn	Bend
1	100	0	0	0	0	0	0	0
2	90	0	0	5	3	0	2	0
3	40	0	0	28	3	0	12	16
4	34	0	0	29	7	0	20	9
5	30	0	0	27	10	0	13	20
6	62	0	0	3	8	0	7	20
7	39	3	0	1	8	0	16	33
8	19	0	0	1	16	0	21	41
9	27	0	0	1	12	0	26	34
10	55	0	0	1	18	0	17	9
11	44	0	0	5	23	0	4	24
12	0	0	0	14	42	0	38	5
13	2	0	0	14	43	0	38	2
14	20	0	0	14	42	0	20	5
15	24	0	0	5	43	0	6	23
16	4	2	0	15	47	0	27	5
17	8	1	0	14	43	0	20	13
18	3	2	0	19	46	0	27	3
19	6	0	0	13	46	0	15	19
20	8	20	0	9	37	0	13	12
21	28	14	0	1	33	0	2	21
22	2	13	0	22	34	0	21	8
23	2	0	0	24	32	0	27	15
24	2	0	0	24	23	0	33	18
25	24	0	0	13	8	0	28	27
26	51	1	0	1	4	0	4	38
27	6	0	0	5	33	0	43	13
28	2	0	0	5	51	0	37	5
29	2	0	0	6	76	0	14	2
30	1	0	0	4	90	0	4	1
31	0	0	0	3	93	0	4	0
32	0	0	0	2	94	0	3	0
33	2	0	0	5	86	0	6	2
34	1	0	0	10	77	0	11	1
35	1	0	1	10	69	0	18	1
36	1	0	1	9	50	0	35	3
37	15	0	0	4	9	0	65	6
38	34	0	0	19	3	0	31	12
39	24	22	1	19	3	0	21	10
40	28	18	1	18	3	0	20	13
41	70	15	1	3	2	0	9	0
42	100	0	0	0	0	0	0	0

Table S7: Backbone hydrogen bond statistics

i+4 --> i

Donor	Acceptor	%age	Dist	Angle
GLY_29@O	GLY_33@N	37%	2.87	157.4
GLU_11@O	GLN_15@N	23%	2.87	157.5
ILE_32@O	VAL_36@N	21%	2.89	161.0
ALA_30@O	LEU_34@N	20%	2.89	157.3
LYS_28@O	ILE_32@N	17%	2.90	163.8
GLY_33@O	GLY_37@N	17%	2.87	152.4
GLN_15@O	PHE_19@N	15%	2.89	161.0
ILE_31@O	MET_35@N	15%	2.89	158.4
ALA_21@O	GLY_25@N	14%	2.85	153.9
LYS_16@O	PHE_20@N	13%	2.89	160.4
LEU_17@O	ALA_21@N	12%	2.89	158.5
SER_26@O	ALA_30@N	10%	2.89	158.1
TYR_10@O	HD2_14@N	10%	2.87	155.7
VAL_12@O	LYS_16@N	10%	2.89	159.4
ASP_7@O	GLU_11@N	7%	2.88	158.8
PHE_20@O	VAL_24@N	7%	2.90	161.6
PHE_19@O	ASP_23@N	7%	2.89	159.0
VAL_18@O	GLU_22@N	7%	2.89	158.7
HID_13@O	LEU_17@N	6%	2.90	160.2
ASN_27@O	ILE_31@N	5%	2.90	161.0
HD2_14@O	VAL_18@N	4%	2.90	161.2
PHE_4@O	SER_8@N	3%	2.89	160.6
GLY_38@O	ALA_42@N	2%	2.89	161.1

i+3 --> i

Donor	Acceptor	%age	Dist	Angle
GLU_11@O	HD2_14@N	17%	2.87	153.7
GLN_15@O	VAL_18@N	12%	2.89	152.4
GLU_3@O	HD1_6@N	11%	2.89	158.4
SER_26@O	GLY_29@N	8%	2.90	152.5
GLU_22@O	GLY_25@N	7%	2.90	153.3
ILE_32@O	MET_35@N	7%	2.90	149.3
ALA_21@O	VAL_24@N	6%	2.91	157.4
LEU_17@O	PHE_20@N	6%	2.90	155.3
LYS_28@O	ILE_31@N	6%	2.90	148.2
GLY_33@O	VAL_36@N	6%	2.91	151.4
GLY_38@O	ILE_41@N	4%	2.91	158.9
LEU_34@O	GLY_37@N	4%	2.91	152.4
GLY_29@O	ILE_32@N	4%	2.90	149.1
MET_35@O	GLY_38@N	4%	2.90	151.6
ILE_31@O	LEU_34@N	3%	2.91	149.4
LYS_16@O	PHE_19@N	3%	2.91	151.3
HD2_14@O	LEU_17@N	3%	2.91	154.7
ASN_27@O	ALA_30@N	3%	2.91	151.1

ASP_23@O	SER_26@N	3%	2.91	156.6
ASP_7@O	TYR_10@N	2%	2.89	148.5

i+5 --> i

Donor	Acceptor	%age	Dist	Angle
GLY_33@O	GLY_38@N	15%	2.87	151.7
LEU_34@O	VAL_39@N	4%	2.90	154.5
ALA_21@O	SER_26@N	2%	2.89	155.3
